# Relationship between Heavy Metal Concentrations in Soils and Grasses of Roadside Farmland in Nepal 

**DOI:** 10.3390/ijerph9093209

**Published:** 2012-09-04

**Authors:** Xuedong Yan, Fan Zhang, Chen Zeng, Man Zhang, Lochan Prasad Devkota, Tandong Yao

**Affiliations:** 1 State Key Laboratory of Rail Traffic Control and Safety, Beijing Jiaotong University, Beijing 100044, China; Email: xdyan@bjtu.edu.cn (X.Y.); 10125479@bjtu.edu.cn (M.Z.); 2 Key Laboratory of Tibetan Environment Changes and Land Surface Processes, Institute of Tibetan Plateau Research, Chinese Academy of Sciences, Beijing 100101, China; Email: zengchen@itpcas.ac.cn (C.Z.); tdyao@itpcas.ac.cn (T.Y.); 3 Central Department of Hydorlogy and Meteorology, Tribhuvan University, Kathmandu 44618, Nepal; Email: devkotalp@hotmail.com

**Keywords:** heavy metals (Cu, Zn, Cd, and Pb), roadside soil, roadside grass, farmland environment, Nepal

## Abstract

Transportation activities can contribute to accumulation of heavy metals in roadside soil and grass, which could potentially compromise public health and the environment if the roadways cross farmland areas. Particularly, heavy metals may enter the food chain as a result of their uptake by roadside edible grasses. This research was conducted to investigate heavy metal (Cu, Zn, Cd, and Pb) concentrations in roadside farmland soils and corresponding grasses around Kathmandu, Nepal. Four factors were considered for the experimental design, including sample type, sampling location, roadside distance, and tree protection. A total of 60 grass samples and 60 topsoil samples were collected under dry weather conditions. The Multivariate Analysis of Variance (MANOVA) results indicate that the concentrations of Cu, Zn, and Pb in the soil samples are significantly higher than those in the grass samples; the concentrations of Cu and Pb in the suburban roadside farmland are higher than those in the rural mountainous roadside farmland; and the concentrations of Cu and Zn at the sampling locations with roadside trees are significantly lower than those without tree protection. The analysis of transfer factor, which is calculated as the ratio of heavy-metal concentrations in grass to those in the corresponding soil, indicates that the uptake capabilities of heavy metals from soil to grass is in the order of Zn > Cu > Pb. Additionally, it is found that as the soils’ heavy-metal concentrations increase, the capability of heavy-metal transfer to the grass decreases, and this relationship can be characterized by an exponential regression model.

## 1. Introduction

High concentrations of heavy metals may affect the ecosystem and human health. Heavy metals present in the roadside soils and grasses may be transported through the food chain to the human body and have a significant toxicity to people. The environmental issues related to heavy-metal contamination are becoming serious in developing countries [1]. With the rapid industrialization and urbanization trend, the increment of traffic activities substantially contributes to the accumulations of heavy metals discharged by vehicles in roadside environments. Heavy-metal pollution in agricultural areas owing to traffic emissions may contaminate the crops growing near the roadways [2]. In agricultural areas, uptake of heavy metals through the soil-crop system could play a predominant role in human exposure to heavy metals [3]. 

Cu, Zn, Cd, and Pb are the typical metal pollutants due to traffic activities [4]. At low doses some heavy metals are essential micronutrients for plants, but in higher doses, they may cause metabolic disorders and growth inhabitation in most plant species [5]. Cu and Zn are trace elements that are essential for human health, but in high doses Cu can cause health problems such as anaemia, liver and kidney damage, and stomach and intestinal irritation [6], and very high levels of zinc can damage the pancreas and disturb the protein metabolism, and cause arteriosclerosis. On the other hand, Cd and Pb, even at extremely low concentrations, are toxic and lead to many diseases, including increased risk of cancer [7]. 

The sources of heavy-metal emissions from vehicles include fuel combustion, lubricating oil consumption, tire wear, brake wear, road abrasion, *etc.* [8,9,10,11,12]. Cd emission is mainly from lubricating oil consumption and tire wear. Zn comes from tire wear and galvanized parts such as fuel tanks [13]. Brake wear is the most important source for Cu and Pb emissions. Pb comes also from exhaust gas and worn metal alloys in the engine [8]. Through the atmospheric deposit or road runoff, heavy metals can be transported into the roadside soils [14,15], where the roadside grasses absorb these heavy-metal elements from the soils through their roots. The grasses’ leaves or stems may also absorb heavy metals from atmospheric particles [16]. On average, the heavy metals’ concentrations in roadside grasses are significantly lower than those in roadside soils [17,18,19]. 

Observation studies have been conducted in many countries to investigate the heavy-metal contamination of roadside soils. It was found that the heavy-metal concentrations were influenced by multiple factors, such as traffic volume [20], highway characteristics [21], road and roadside terrain [22], roadside distance, wind direction [23], rainfall [20], seeded strip [17,18,19,20,21,22,23,24], local economy [25,26], *etc*. High traffic volume can heighten the heavy-metal content in the roadside soils [20]. Correspondingly, the roadside heavy-metal concentrations in developed areas are higher than those in underdeveloped areas [22]. It has been reported that the heavy-metal content has a belt-shaped distribution in terms of roadside distance, decreasing exponentially with the distance [27]. The influential scope of traffic pollution in roadside soil can be up to 100 m from the road edge [28], and most of the deposited metal particles remain in the 0–5 cm of the roadside topsoil [24]. Through absorption from roadside soils, the heavy-metal elements are further transferred into the roots, stem, and leaves of grasses. Therefore, the factors influencing the soil’s heavy-metal content have nearly the same effect on the heavy-metal concentrations in roadside grass.

In order to investigate the relationship between the heavy-metal content of soils and corresponding grasses, the Transfer Factor (TF), defined as the ratio of heavy-metal concentration in grass to that in soil, has been applied to assess plants’ capability to absorb heavy metals from the soil [18,19,20,21,22,23,24,25,26,27,28,29]. TF is also called bio-concentration factor (BCF), calculated as the ratio of element concentration in plant tissues to the element concentration in the soil [30]. It should be noticed that only when a linear relationship is observed between the concentrations of grass and soil for a given element, TF can be an appropriate measure for the assessment [31]. It was found that soil properties, grass absorption ability, and both the form and concentration of heavy metals in the soil comprehensively impact the uptake capability of heavy metals from the soil by the corresponding local grass [29,31]. It was observed that Zn has the highest TF value, followed by Cu and Cd, and Pb has the lowest TF value. Even when the samples are mixed with different types of grasses, a similar pattern can still be observed [29]. However, few studies were focused on the roadside farmland environments to explore the relationship between the heavy-metal contaminations in soil and corresponding grass resulting from traffic activities. 

Previous environmental studies in the Kathmandu area have indicated that the air pollutants and heavy metal contaminants in tree leaves are substantially due to the city’s traffic emissions [32,33]. The authors of this paper have conducted a study to investigate the influence of transportation activities on farmland soils along a highway across mountainous areas around Kathmandu in Nepal [34]. It was found that concentration patterns of the heavy metals in soil proved their homology with the traffic pollution source. Furthermore, some spots with peak concentrations may be severely polluted although average accumulations of heavy metals pose no hazard in the region. To ensure food safety and ecosystem security, the forms and behavior of heavy metals in farmland need to be dynamically monitored and assessed [35]. The heavy-metal transport process in roadside grasses is the same as that in crops. Therefore, this research aims at characterizing the effect of location, roadside distance, and tree protection on heavy-metal distributions in roadside farmland soils and corresponding grasses, using the MANOVA experimental design method. Especially, the objective of this study is to explore the relationship between heavy-metal concentrations in the roadside soils on the heavy-metal uptake capabilities of the corresponding local grasses.

## 2. Material and Method

### 2.1. Site Description

**Figure 1:**
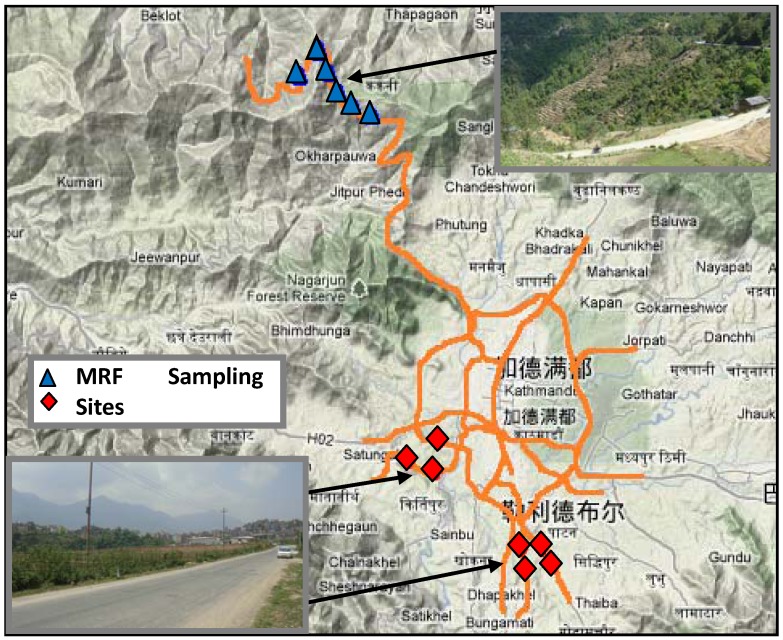
Sampling sites around Kathmandu, Nepal.

The grass and corresponding soil samples were collected in the roadside farmland along the Trishuli Highway and suburban roadways around Kathmandu, Nepal (See Figure 1). The Kathmandu Metropolitan City is the capital of Nepal, which is also Nepal’s political, economical and cultural center. Kathmandu is located in a valley within the Lesser Himalaya at an altitude of 1,350 meters, with almost 990,000 people. The Trishuli Highway connecting the Trishuli City and Kathmandu is a 4.5 m wide gravel/dirt track with a 40 mph design speed and low traffic volume. Due to the Trishuli Highway across the mountain area, the roadside farmland is terraced but closely connected to the road edge. The suburban roads are urban minor roads and the average width is 7 meters. It is clear that the level of traffic volume in urban minor roads is higher than that on the Trishuli Highway and the anthropogenic influence on environments in the suburban area is more intense than that in the mountain area. Additionally, unleaded gasoline was introduced in Kathmandu in July 1997, and distributed throughout Nepal since 1999 [36].

### 2.2. Factorial Experiment Design

According to the features of the study site, the experiment follows a 2 × 2 × 2 × 5 MANOVA design for analyzing whether the independent variables sampling site, roadside tree and distance have a statistically significant effect on the concentration distribution of Cu, Zn, Cd, and Pb in the roadside farmland soil and corresponding grasses. Table 1 shows the detailed description of the four independent variables. Location is defined as whether the sampling site is in rural mountainous roadside farmland (MRF) or suburban roadside farmland (SRF). Sample type (GorS) indicates whether the heavy-metal concentration data are from Grass or Soil. Distance is defined as the roadside distance perpendicular to the road edge. The sampling distances to the road edge are designed as 0 m, 10 m, 30 m, 50 m, and 100 m. Since trees intermittently grow along the Trishuli Highway and the suburban minor roads, whether the trees have a protection effect on the heavy-metal pollution was also investigated.

**Table 1:** Description of four independent variables for MANOVA.

Independent Variable	Variable Definition	Discrete Level
Location	Whether the sampling site is in mountain area or suburban area	Level 1: Mountainous roadside farmland (MRF)
		Level 2: Suburban roadside farmland (SRF)
Sample type (GorS)	Whether the heavy-metal concentration data are from grass sample or soil sample	Level 1: Grass sample
		Level 2: Soil sample
Distance	The roadside distance from the soil sampling location perpendicular to the road edge	Level 1: 0 m
		Level 2: 10 m
		Level 3: 30 m
		Level 4: 50 m
		Level 5: 100 m
Tree	Whether there are trees growing along the road edge or not	Level 1: Tree—Trees exist between farmland and roads and the trees’ continues distance is at least 30 m along the road direction
		Level 2: No Tree—There are no trees between farmland and road

### 2.3. Grass and Soil Sampling

The relationship between soil samples and grass samples is a one-to-one correspondence relationship. As shown in Table 2, a total of 60 grass and 60 topsoil samples were collected under a dry weather conditions, where 26 grass and 26 topsoil samples were taken along the Trishuli Highway and 34 grass and 34 topsoil samples were taken along the suburban roads. The topsoil samples are in depth of 0–5 cm and the grass samples are stems and leaves of the roadside grasses. Considering universality and uniformity, several grasses that were common in the field were collected, including *Taraxacum mongolicum* Hand.-Mazz, *Cirsium setosum* (Willd.) MB., and *Bidens pilosa* Linn. All of the grasses belong to the same family, Asteraceae.

**Table 2:** Soil-grass sample size distribution by Location × Distance × Tree.

Location	Tree	Distance					Total
		0 m	10 m	30 m	50 m	100 m	
MRF	No tree	2–2	4–4	4–4	4–4	3–3	17–17
	Tree	2–2	2–2	2–2	2–2	1–1	9–9
SRF	No tree	3–3	3–3	3–3	3–3	3–3	15–15
	Tree	4–4	4–4	4–4	4–4	3–3	19–19
Total		11–11	13–13	13–13	13–13	10–10	60–60

**Table 3:** Descriptive statistical results of heavy-metal concentrations (mg/kg) in soil and grass.

Factor	Level	Cu (Grass)		Cu (Soil)		Zn (Grass)		Zn (Soil)		Cd (Grass)		Cd (Soil)		Pb (Grass)		Pb (Soil)	
		Mean	S.D.	Mean	S.D.	Mean	S.D.	Mean	S.D.	Mean	S.D.	Mean	S.D.	Mean	S.D.	Mean	S.D.
Location	MRF	11.46	3.88	25.39	6.03	35.15	10.90	92.09	38.07	0.24	0.28	0.23	0.30	4.51	4.63	20.96	10.23
	SRF	11.92	4.15	27.91	7.43	59.70	28.80	84.46	33.38	0.27	0.37	0.30	0.29	3.73	2.67	31.81	10.70
Distance	0 m	11.15	2.93	25.89	7.04	62.57	38.20	86.00	32.39	0.25	0.36	0.34	0.24	5.57	3.62	28.57	13.19
	10 m	11.15	5.48	27.05	8.53	46.67	13.90	87.13	48.39	0.27	0.35	0.27	0.22	3.07	2.26	27.18	12.03
	30 m	12.00	3.94	26.98	6.67	47.55	33.10	77.91	19.19	0.23	0.27	0.21	0.14	4.47	5.12	27.44	13.18
	50 m	11.73	3.01	27.33	6.12	44.38	13.60	87.88	29.40	0.24	0.34	0.28	0.39	3.03	1.48	26.25	11.37
	100 m	12.57	4.69	26.68	7.27	45.49	21.45	101.75	43.40	0.30	0.40	0.24	0.15	4.41	4.47	26.20	10.57
Tree	No tree	12.67	3.74	29.32	5.43	47.97	30.40	97.87	33.23	0.16	0.20	0.27	0.21	4.08	4.31	27.59	11.33
	Tree	10.56	4.08	23.76	7.39	50.40	19.06	75.42	34.55	0.37	0.42	0.27	0.28	4.05	2.68	26.53	12.41
Total		11.72	4.01	26.82	6.92	49.06	25.77	87.77	35.38	0.26	0.33	0.27	0.24	4.07	3.64	27.11	11.74

### 2.4. Sample Processing

Every soil sample was air-dried in the lab, pulverized with an agate mortar, and then sieved through a nylon sieve with a pore diameter of ≤0.149 mm. Next, 0.3 ± 0.0001 g sieved soil sample was weighed each time, put in a tetrafluoroethylene (PTFE) beaker, mixed with 6 mL HNO_3_-3 mL HCl-0.25 mL H_2_O_2_, and then heated in microwave digestion system (GEM mars). The microwave digestion settings are listed in Table 3. After that, the digested solution was diluted to 50 mL with ultra-pure water and filtered through a 0.45 μm microporous membrane. Finally, 1.0 mL filtered solution was diluted to 10 mL for measurement of Pb, Cd, Cu and Zn by Inductively Coupled Plasma-Mass Spectrometry (ICP-MS, Thremo X Series 2). 

Plant samples were dried by drying oven and pulverized with a pulverizer. Then, a 1.0 g plant sample was weighted in a digestion pipe and mixed with 1 mL H_2_SO_4_-8 mL HNO_3_-1 mL HClO_4_. After that, the digestion pipe was covered with a funnel and heated in graphite furnace over low temperature till the sulfuric acid digestion smoked. If there was any black residue, 0.5 mL HCLO_4_ was added and the digestion pipe was heated again. The operation was repeated until the digested solution became clear. Finally, the digested solution was filtered through a 0.45 μm microporous membrane and diluted to 100 mL for measuring Pb, Cd, Cu and Zn by Inductively Coupled Plasma-Mass Spectrometry (ICP-MS, Thremo X Series 2). 

For the processing of both soil and plant samples, a series of standard samples were also prepared for the purpose of quality control, including: (1) analyzing 14 random samples, one blank sample and 1 standard sample each time; and (2) randomly selecting samples to ensure that the relative standard deviation were less than about 10%.

## 3. Results and Discussion

### 3.1. Descriptive Statistics of Heavy-Metal Concentrations in Roadside Soil and Corresponding Grass

The statistical description of heavy-metal concentrations (mg/kg) in soil and grass under three independent factors are summarized in Table 3. On average, the heavy-metal content of grass and soil in MRF are consistently lower than those in SRF, except for Zn (Soil) and Pb (Grass). Compared to the further roadside distances, the road edge concentrations of Zn in Grass, Cd in Soil, Pb in Grass and Pb in Soil are higher due to traffic emission. However, as the roadside distance increases, there is no constant decreasing pattern in heavy-metal content distribution presumably because the frequent farming activities such as irrigation, plough, and fertilization may mix the farmland top soil spatially and disturb the roadside heavy metal distance-distribution pattern [34]. Additionally, the heavy-metal concentrations in either soil or grass under the tree protection are generally lower than those without the tree protection, except for Cd.

### 3.2. Correlation Analysis of Heavy-Metal Concentrations

Table 4 summarizes the descriptive statistical results of Transfer Factor (TF) of heavy metal from soil to grass, which is calculated as the ratio of heavy-metal concentrations in grass to those in the corresponding soil. According to the previous research results [29,31,32,33,34,37], TF value should be below 1. However, the observation in this study shows a mean value of Cd’s TF of 1.35, which indicates that the concentration of Cd in grass is higher than that in soil. It can be inferred that the soil is not the only source for contamination of Cd in grass that may absorb the heavy metal from air deposition [31] or other unknown sources. Additionally, it was found that Cd can be easily absorbed by plants [38]. Excluding Cd from the analysis of TFs, the order of uptake capability from soil to grass is Zn > Cu > Pb. This order is the same as the previous study results for the unique plants [31,32,33,34,37,39]. It can be concluded that even though the types of grasses are different, the order for heavy-metal absorbing capability from soil to grass is similar. Furthermore, the factors of Location, Distance, and Tree do not show a consistent influence on TF for the four types of heavy metals. 

**Table 4:** Descriptive statistical results of transfer factor of grass-soil heavy-metal concentrations.

Factor	Level	Cu-TF		Zn-TF		Cd-TF		Pb-TF	
		Mean	S.D.	Mean	S.D.	Mean	S.D.	Mean	S.D.
Location	MRF	0.47	0.18	0.44	0.24	1.19	1.10	0.25	0.30
	SRF	0.44	0.18	0.79	0.41	1.47	2.26	0.12	0.76
Distance	0 m	0.45	0.13	0.80	0.47	0.85	1.01	0.23	0.17
	10 m	0.41	0.17	0.63	0.28	1.31	1.59	0.12	0.10
	30 m	0.46	0.17	0.64	0.45	1.16	1.11	0.23	0.40
	50 m	0.45	1.36	0.60	0.36	1.51	1.79	0.15	0.11
	100 m	0.52	0.28	0.54	0.35	1.90	3.19	0.15	0.10
Tree	No tree	0.45	0.18	0.56	0.43	1.05	1.92	0.18	0.27
	Tree	0.46	0.19	0.74	0.29	1.70	1.71	0.17	0.10
Total		0.46	0.18	0.64	0.38	1.35	1.84	0.18	0.21

Table 5 summarizes the correlation analysis of Cu, Zn, Cd, and Pb concentrations based on the combined data of soil and grass. It shows that Cu, Zn, and Pb are significantly correlated with each other while Cd has no correlation with Cu and Pb. The correlation pattern indicates that the heavy-metal concentrations of Cu, Zn, and Pb in roadside soil and grass are associated with traffic contamination but Cd might be influenced by other unknown sources. 

**Table 5:** Correlation Analysis of Cu, Zn, Cd, and Pb Concentrations based on the combined data of soil and grass.

	Cu	Zn	Cd	Pb
Cu	1.000	0.588 *	−0.012	0.770 *
Zn	0.588 *	1.000	0.269 *	0.451 *
Cd	−0.012	0.269 *	1.000	0.088
Pb	0.770 *	0.451 *	0.088	1.000

* Correlation is significant at the 0.01 level (2-tailed).

Table 6 summarizes the further correlation analyses of the four heavy metals in soil and grass respectively. The soil’s heavy-metal correlation analysis shows a similar trend displayed in Table 6 as the combined data, except that Pb is not correlated with Zn any more. However, among the heavy metals in grass, the only significant correlation exists between Cu (Grass) and Zn (Grass). The correlation analyses indicate that (1) the transportation activities are the major heavy-metal pollution source to each data collection sites; (2) Since the heavy metals directly accumulate into the roadside soils while the grasses partly absorbs heavy metals from the soils, more significant correlations among the heavy metals can be identified in soil than those in grass; (3) based on the previous TF analysis, different capabilities that grass absorbs Cu, Zn, Cd, and Pb from soil may result in the weak correlation among the heavy metals in the grass samples.

**Table 6:** Correlation Analyses of Cu, Zn, Cd, and Pb Concentrations for Soil and Grass Samples, Respectively.

	**Cu (Soil)**	**Zn (Soil)**	**Cd (Soil)**	**Pb (Soil)**
Cu (Soil)	1.000	0.339 **	0.009	0.460 **
Zn (Soil)	0.339 **	1.000	0.554 **	0.045
Cd (Soil)	0.009	0.554 **	1.000	0.153
Pb (Soil)	0.460 **	0.045	0.153	1.000
	**Cu (Grass)**	**Zn (Grass)**	**Cd (Grass)**	**Pb (Grass)**
Cu (Grass)	1.000	0.267 *	−0.121	−0.171
Zn (Grass)	0.267 *	1.000	0.089	0.070
Cd (Grass)	−0.121	0.089	1.000	0.154
Pb (Grass)	−0.171	0.070	0.154	1.000

** Correlation is significant at the 0.01 level (2-tailed); * Correlation is significant at the 0.05 level (2-tailed).

**Table 7:** MANOVA of heavy-metal concentrations.

Source	df	Cu		Zn		Cd		Pb	
		F	Sig.	F	Sig.	F	Sig.	F	Sig.
Location	1	5.591	0.020 *	3.439	0.067	0.238	0.627	12.238	0.001 *
Tree	1	19.364	0.000 **	4.169	0.044 *	3.457	0.066	1.617	0.207
Distance	4	0.079	0.989	0.537	0.709	0.124	0.974	0.379	0.823
GorS	1	215.352	0.000 **	51.252	0.000 **	0.014	0.907	206.181	0.000 **
Location × Tree	1	2.298	0.133	0.700	0.405	0.012	0.912	1.657	0.201
Location × Distance	4	0.478	0.752	0.237	0.917	0.893	0.471	0.703	0.592
Location × GorS	1	1.969	0.164	6.239	0.014 *	0.443	0.507	15.822	0.000 **
Tree × Distance	4	0.665	0.618	1.072	0.375	0.555	0.696	0.301	0.877
Tree × GorS	1	4.137	0.045 *	2.852	0.094	4.474	0.037 *	1.209	0.274
Distance × GorS	4	0.163	0.957	0.829	0.510	0.264	0.900	0.082	0.988

* Correlation is significant at the 0.05 level (2-tailed); ** Correlation is significant at the 0.01 level (2-tailed).

### 3.3. MANOVA of Heavy-Metal Concentrations

In the subsequent statistical analyses using the combined data of soil and grass samples, a MANOVA is applied to investigate differences between different levels of the four factors (see in Table 7). The hypothesis testing in the following analysis is based on a 0.05 significance level. The result indicates that the independent factors are complicatedly associated with the concentrations of Cu, Zn, and Pb. Location influences the concentrations of Cu (*P* = 0.02) and Pb (*P* = 0.001). Tree influences the concentration of Cu (*P* < 0.001) and Zn (*P* = 0.044). GorS have a significant effect on the concentration of Cu (*P* < 0.001), Zn (*P* < 0.001) and Pb (*P* < 0.001). Furthermore, GorS shows a significant interaction effect with Location on Zn (*P* = 0.014) and Pb (*P* < 0.001) and interaction with Tree on Cu (*P* = 0.045) and Cd (*P* = 0.037). However, roadside distance does not significantly influence any heavy metal’s concentration. Additionally, there are almost no factors influencing Cd concentration. The Cd’s distribution and concentration in Kathmandu region might be affected by some other unknown sources.

#### 3.3.1. Effects of Independent Factors on Heavy-Metal Concentrations

**Figure 2:**
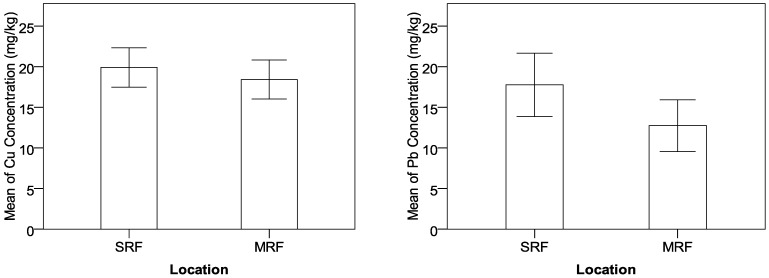
Effects of location on concentrations of Cu and Pb.

Figure 2 displays the effects of Location on concentrations of Cu and Pb. Clearly, the mean values of Cu and Pb concentrations in the suburban roadside farmland (SRF) are higher than those in the rural mountainous roadside farmland (MRF). As mentioned early, the traffic volume in urban minor roads is higher than that in the rural Trishuli Highway and the anthropogenic influence on environments in the suburban area is more intense than that in the mountain area, which contributes to more heavy-metal accumulations in suburban area than rural mountain area. The finding is consistent with previous research conclusion that local economy and land use type lead to variation in heavy-metal distribution [25,26].

**Figure 3:**
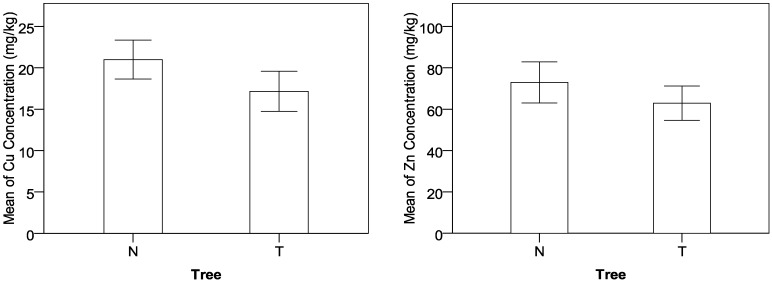
Effects of tree protection on concentrations of Cu and Zn.

It is found that roadside trees show a remarkable positive effect on the control of heavy-metal concentrations, and concentrations of Cu and Zn at the sampling locations with roadside trees are significantly lower than those without tree protection, as shown in Figure 3. Planting trees can effectively prevent the pollution particles from depositing to roadside farmland so that more heavy-metal contaminants can be expelled into drainage facilities. In addition, trees can be used to remove, transfer, or stabilize heavy-metal soil contaminants to render them harmless [40]. In recent studies, phyto-remediation has been considered as a promising new countermeasure for *in situ *cleanup of heavy-metal contaminated soils [41,42].

**Figure 4:**
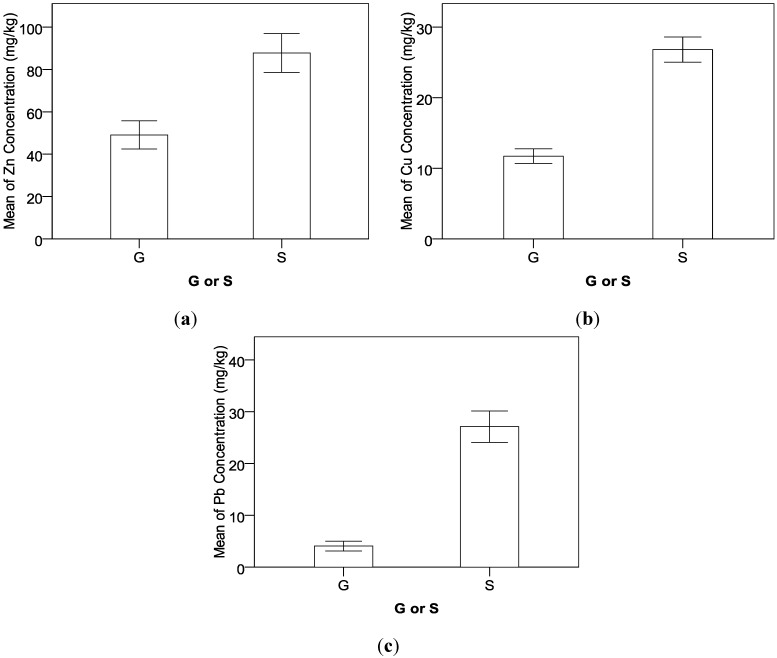
Difference of heavy-metal concentrations between grass and soil samples.

Figure 4 displays the difference of heavy-metal concentrations between grass and soil samples. Based on the TF analysis, the order of TF that indicates the uptake capability from soil to grass is Zn > Cu > Pb. Being consistent with the TF analysis, the Zn concentration in grass is more than half of the concentration in soil, the Cu concentration in grass is between one third and half of the concentration in soil, and the Pb concentration in grass is only about one sixth of the concentration in soil.

#### 3.3.2. Interaction Effect between Factors on Heavy-Metal Concentrations

**Figure 5:**
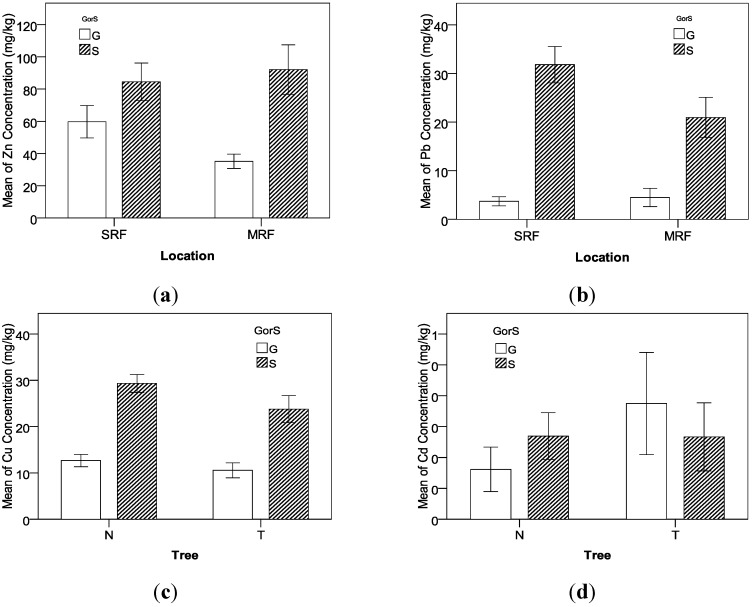
Interaction effects between GorS, location, and tree on heavy-metal concentrations.

As shown in Table 7 and Figure 5, all interaction effects between factors involve GorS. The interaction effect between GorS and Location on Zn shows that compared to the samples in the suburban roadside farmland (SRF), the soil’s Zn concentration in rural mountain roadside farmland (MRF) is slightly higher (Figure 5(a)). Zn accumulation in soil owing to vehicle emission is mainly from engine oil, tire wear, and brake wear [13]. Because the highways in mountain areas have more vertical and horizontal curves than the roadways in the flat suburban area, it can be expected the vehicles’ braking, accelerating, and steering behaviors are much more frequent and intense in the mountain highway than the flat suburban roadways. Therefore, although there the traffic volume of suburban roadways is higher, their roadside Zn accumulation is slightly lower than that of the mountain highway. However, the grasses’ Zn concentration in the rural mountain area is significantly lower than that in the suburban area. The possible reason is that the complex terrain characteristics in the mountain highway can cause higher local roadside wind frequency and intensity than the suburban roadway, which may reduce the capability that roadside grass’s stem and leaves absorb Zn in the air directly.

The interaction effect between GorS and Location on Pb shows that the soil’s Pb concentration in rural mountain roadside farmland (MRF) is significantly lower compared to the soil samples in the suburban roadside farmland (SRF); however there is no obvious difference in the grasses’ Pb concentration between MRF and SRF (Figure 5(b)). Because gas consumption and worn metal alloys in the vehicle engine are the major sources of roadside soil Pb accumulation [8], the higher traffic volume in suburban roadways leads to higher heavy-metal content in SRF [20]. However, grasses may have a self-protection mechanism that resists the uptake of heavy metals from soils with high concentrations.

The interaction effect between GorS and Tree on Cu shows that the roadside tree’s protection effect on Cu accumulation in soil is better than that in grass (Figure 5(c)). However, the interaction effect between GorS and Tree on Cd displays that the grasses’ Cd concentration is higher with roadside trees compared to without trees while there is no significant difference in soils’ Cd concentration between the samples with and without roadside trees (Figure 5(d)). As explained before, the Cd accumulation in data collection sites might be disturbed by other unknown sources. For example, a longer tillage history can lead to a high level of heavy metal contamination for cultivated soils [35].

### 3.4. Relationship between Soil-to-Grass TF and Heavy-Metal Concentration in Soil

In order to investigate the relationship between capabilities that grass absorbs heavy metal from soil (named TF, the ratio of heavy-metal concentration in grass to that in soil) and soil’s heavy-metal concentrations, we conduct a series of regression analyses for the two variables. Four types of regression forms were considered for this investigation, including:

■ Linear Model: *Y* = *a* + *b × X*
■ Quadratic Model: *Y* = *a* + *b* ×*X* + *c*×*X*^*2*^
■ Logarithmic Model: *Y* = *a* + *b* × ln(*X*)
■ Exponential Model: *Y* = *a* + *b*^*X*^

where Y represents TF and X represents the value of heavy-metal concentration in soil. 

**Figure 6:**
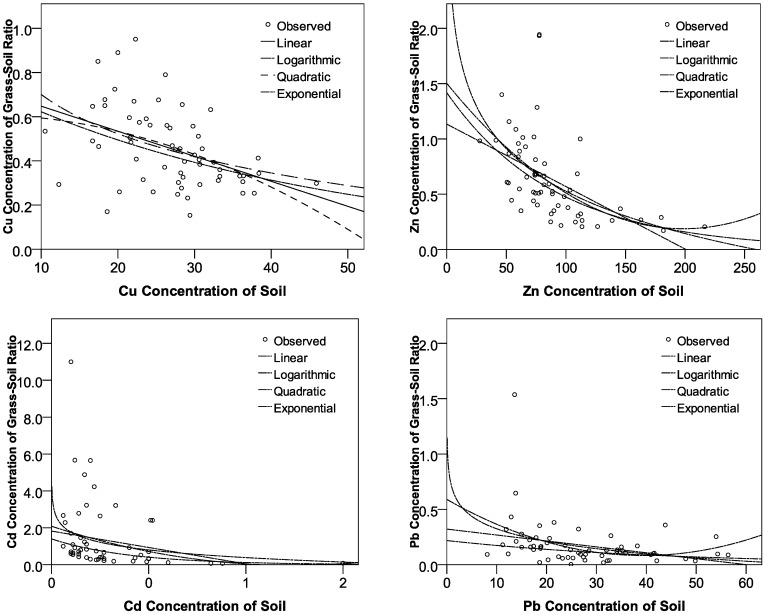
Relationship between the ratio of heavy-metal concentration in grass to soil and heavy-metal concentrations in soil.

Figure 6 and Table 8 show that the TFs of Cu, Zn, Cd, and Pb have monotone decreasing relationships with the corresponding heavy-metal concentrations. This interesting phenomenon indicates that the higher the heavy-metal concentrations in soils, the lower the capabilities of grasses to absorb heavy metals from soils. The previous biological study concluded that grasses have a range of potential mechanisms at the cellular level that might be involved in the detoxification and their tolerance to heavy-metal stress including roles in reduced absorption or efflux pumping of metals at the plasma membrane [43]. It implies that the grasses’ self-protection mechanism would contribute to mitigating uptake of heavy metals from soils at high concentration sites. However, the above finding cannot be explained as that a higher soil’s heavy-metal concentration results in a lower grasses’ heavy-metal concentration. The TF only indicates a capability of grass absorbing heavy metals, but not an absolute value of heavy-metal content. Furthermore, Table 8 summarizes the four models’ parameters and goodness-of-fit statistics. Almost all of the four models for the four metals correspondingly can significantly characterize the relationship between TF and heavy-metal concentrations in soil, except for the linear model, quadratic model, and logarithmic model for Cd, which have *P*-values larger than 0.05.

**Table 8:** Models’ parameters and goodness-of-fit statistics.

Heavy Metals	Equation	Model Summary					Parameter Estimates		
		R^2^	F	df1	df2	Sig.	a	b	c
Cu	Linear	0.189	13.5	1	58	0.001	0.761	−0.011	--
	Logarithmic	0.159	10.9	1	58	0.002	1.29	−0.256	--
	Quadratic	0.196	6.94	2	57	0.002	0.612	9.538E−5	−2.119E−4
	Exponential	0.157	10.8	1	58	0.002	0.783	−0.023	--
Zn	Linear	0.272	21.7	1	58	0.000	1.134	−0.006	--
	Logarithmic	0.288	23.5	1	58	0.000	3.076	−0.553	
	Quadratic	0.3	12.2	2	57	0.000	1.503	−0.013	3.33E−5
	Exponential	0.449	47.3	1	58	0.000	1.419	−0.011	--
Cd	Linear	0.055	3.38	1	58	0.071	1.822	−1.777	--
	Logarithmic	0.057	3.51	1	58	0.066	0.387	−0.599	--
	Quadratic	0.064	1.94	2	57	0.154	2.084	−3.427	1.387
	Exponential	0.287	23.3	1	58	0.000	1.398	−2.462	--
Pb	Linear	0.087	5.5	1	58	0.022	0.322	−0.005	--
	Logarithmic	0.114	7.48	1	58	0.008	0.7	−0.163	--
	Quadratic	0.145	4.83	2	57	0.012	0.59	−0.026	3.365E−4
	Exponential	0.078	4.94	1	58	0.030	0.218	−0.023	--

## 4. Conclusions

This study conducted a MANOVA experiment to investigate the heavy-metal concentrations in roadside soils and corresponding grasses in roadside farmland around Kathmandu, Nepal. The sampling location, roadside distance, tree protection, and the type of samples are selected as the tested factors. 

Based on the correlation analyses of heavy metals in both grass and soil samples, it was found that more significant correlations among the heavy metals can be identified in soil samples than those in grass samples. This is because the uptake capabilities from soil to grass for different heavy metals are significantly different.

The MANOVA results indicate that the concentrations of Cu, Zn, and Pb in the soil samples are significantly higher than those in the grass samples. The TF order indicating uptake capability from soil to grass is Zn > Cu > Pb. Because the order is the same as the previous studies for the unique plants, it implies that the TF order of Cu, Zn, and Pb would be applicable for different types of grasses. It was also found that the concentrations of Cu and Pb in the suburban roadside farmland are higher than those in the rural mountainous roadside farmland because of the difference in traffic volume between the two types of sampling locations. Additionally, the factor of Tree influences the concentrations of Cu and Zn. The analysis indicates that the trees growing linearly along the roadways can effectively reduce the heavy metals’ concentrations in the roadside farmland. Therefore, planting trees may be considered as an effective remedy countermeasure for the existing crop plots that are very close to roadways.

The most interesting finding in this study is that the transfer factor influencing the capability that grass absorbs heavy metal from soil is related to the soil’s heavy-metal concentrations. There is a monotone decreasing relationship between transfer factor and heavy-metal concentrations in soil: as the soil’s heavy-metal concentrations increase, the grass’s capability of heavy-metal absorption decreases. The exponential regression form is the best model that can characterize this relationship statistically. The finding implies that the grass’s self-protection mechanism would mitigate grasses’ uptake of heavy metals from soil at high concentration sites.

The results of this study would be useful for understanding how the heavy-metal accumulations in farmland roadside soils and grasses owning to traffic activities are influenced by roadside attributes, which is helpful in making policies for avoiding heavy-metal contaminants in agricultural areas. Furthermore, the relationship between transfer factor and the ratio of heavy-metal concentration in grass to that in soil deserves a further lab experiment study under well-controlled settings to accurately describe the influence mechanism of heavy-metal concentration in the roadside soil on the heavy-metal uptake capabilities of the corresponding local grasses.
